# Re-examining the testing effect as a learning strategy: the advantage of retrieval practice over concept mapping as a methodological artifact

**DOI:** 10.3389/fpsyg.2023.1258359

**Published:** 2023-12-21

**Authors:** Roland Mayrhofer, Christof Kuhbandner, Katja Frischholz

**Affiliations:** Department of Psychology, University of Regensburg, Regensburg, Germany

**Keywords:** testing effect, retrieval practice, concept mapping, learning strategies, educational psychology, cognitive psychology

## Abstract

Several previous studies appear to have demonstrated that studying with retrieval practice produces more learning than studying with concept mapping, a finding based on which an extended use of retrieval practice in educational practice was recommended. However, a closer examination of the methods used in these previous studies reveals a crucial confounding variable: Whereas participants in the concept mapping conditions performed a concept mapping task without any subsequent memorizing of the learning material, participants in the retrieval practice conditions performed not only retrieval practice but also an additional memorization task, which doubled the total memorization time. The present preregistered study examined whether the advantage observed in the retrieval practice condition over the concept mapping condition in previous studies was actually driven by additional memorization rather than by retrieval practice. While we replicated the previous finding that retrieval practice in combination with additional memorizing produces more learning than concept mapping without additional memorizing, this advantage of retrieval practice over concept mapping vanished when participants in the concept mapping condition, too, memorized the learning material after having created a concept map. These findings demonstrate that the assumed advantage of retrieval practice over concept mapping in fact represents a methodological artifact. Besides serving as a reminder of the importance of a solid methodology, the present study also illustrates the importance of using of an adequate terminology. Depicting a learning strategy condition as “retrieval practice” when the condition actually encompasses not only retrieval practice but also additional memorizing obfuscates the possibility that observed memory advantages may not be fueled by retrieval practice, i.e., the learning strategy as such. We conclude by giving an outlook on the ramifications of our findings for cognitive and educational psychology.

## 1 Introduction

The effectiveness of learning strategies is an important topic that is extensively researched in applied research. In a highly prominent and frequently cited study, Karpicke and Blunt (2011) investigated in the context of learning strategies an important finding from basic research, namely, the so-called testing effect, which describes the phenomenon that retrieval enhances long-term memory. They came to the conclusion that retrieval practice produces more learning than elaborative studying using concept mapping. A virtually identical result was found by O’Day and Karpicke (2021), who employed the same methodology as Karpicke and Blunt (2011). These results were also found and therefore confirmed by Lechuga et al. (2015) and Camerer et al. (2018), who also employed the same methodology as Karpicke and Blunt (2011), although the advantage of retrieval practice was notably smaller than compared to the work of Karpicke and Blunt (2011). In the light of the far-reaching ramifications for both cognitive and educational psychology if in fact retrieval practice really does produce more and better learning than elaborative studying with concept mapping, it is evidently important to ascertain that the basis for such propositions is theoretically and methodologically solid. This is why this study re-examined and empirically tested the proposition that retrieval practice produces more learning than elaborative studying with concept mapping, focusing primarily on the methodology of previous experiments.

Karpicke and Blunt (2011) as well as O’Day and Karpicke (2021) conclude that retrieval practice is a better learning strategy because they report to have empirically shown that retrieval practice produces more learning than elaborative studying with concept mapping. Specifically, their conclusion is based on their finding that performance in a memory test was better in a retrieval practice condition compared to a concept mapping condition. We propose, however, that the reasons for the better performance in the retrieval practice condition, as found by Karpicke and Blunt (2011) and O’Day and Karpicke (2021), and, by extension, also in the studies by Lechuga et al. (2015) and Camerer et al. (2018), which employ the same methodology, are not based on certain specific cognitive mechanisms inherent to or associated with the respective learning strategy. Instead, there is reason to assume that the better performance in the retrieval practice condition occurred due to a methodological artifact because a closer analysis of the methods employed by Karpicke and Blunt (2011) reveals two potential confounders inherent in the design and execution of these studies, which might have biased the observed results. These potential confounders also affect the studies by Lechuga et al. (2015), Camerer et al. (2018), and O’Day and Karpicke (2021).

**FIGURE 1:**
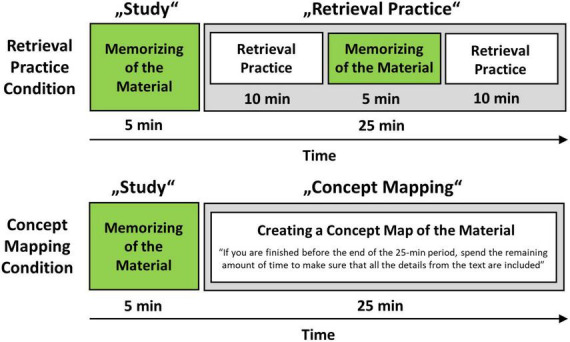
Illustration of the confounding variable “Memorization” in Karpicke and Blunt’s (2011) study. The terms used to describe the different learning strategy conditions, namely, “retrieval practice” and “concept mapping,” give the impression that only retrieval practice or concept mapping, respectively, were performed in each condition. However, in the so-called “retrieval practice” condition, participants not only performed a retrieval task but also an additional memorization task. By contrast, in the concept mapping condition, participants only performed a concept mapping task without any additional memorization of the learning material. The additional memorization task doubled the time participants spent memorizing the learning material for the later test in the retrieval practice condition. Note that the text the participants were to learn was available during the creation of the concept map but not during retrieval practice.

The first potential confounder pertains to Karpicke and Blunt’s (2011) operationalization of what they refer to as “retrieval practice.” At the beginning of their experiment, participants in all conditions were asked to study a text about sea otters for a 1-week delayed memory test for 5 min. After that, conditions differed, but a closer look reveals that the conditions differed not only—as the designation as “retrieval practice” and “concept mapping” suggests—in that the participants performed retrieval practice in one condition and concept mapping in the other. Rather, in the “retrieval practice” condition, participants performed a memorization task in addition to retrieval practice, as illustrated below in Figure 1. In this memorization task, they were asked to memorize the text for 5 min. By contrast, in the concept mapping condition, participants only created their concept map, and there was no additional memorization task. In particular, participants in the concept mapping condition were instructed not to invest any additional time in memorizing the material as they were told “that if they finished before the end of the 25-min period, they should spend the remaining amount of time reviewing their map and making sure they had included all the details from the text in their map” (Karpicke and Blunt, 2011).

This difference between conditions, when there is an additional memorization phase in one condition but not in the other, is problematic for at least three reasons. First, from a methodological perspective, it seems likely that the advantage observed by Karpicke and Blunt (2011) in the retrieval practice condition over the concept mapping condition was not actually driven by retrieval practice but rather by the additional memorization phase, which doubled the time the participants had in the retrieval practice condition to memorize the learning material for the later test.

There is substantial evidence reaching back as early as Ebbinghaus (1913) that learning performance increases with increased memorization time (Murdock, 1960; Bugelski, 1962; Zacks, 1969; Geiselman, 1977; Fredrick and Walberg, 1980; Gettinger, 1991; Cook et al., 2010; Yang et al., 2016; Chen and Yang, 2020). This indicates, roughly speaking, that learning performance increases with increased memorization time.

Furthermore, the difference in memorization time between the conditions might also act as confounder in another way. According to the well-established spacing effect (e.g., Rohrer and Pashler, 2007; De Jonge et al., 2012; Kim et al., 2019; Murphy et al., 2022), distributed learning is more effective than massed learning. Therefore, considering that in the retrieval practice condition in Karpicke and Blunt’s (2011) study participants memorized the learning material in two study phases at different time points during the experiment (in the initial study phase and the subsequent retrieval practice phase, see Figure 1), this condition represents an example for distributed learning. By contrast, in the concept mapping condition, participants memorized the learning material in only one study phase (in the initial study phase), which represents massed learning. This could indicate that the spacing effect may additionally have contributed to the observed advantage in the retrieval practice condition. This is further supported by a demonstration that spacing also affects the testing effect, as reported by Carpenter and DeLosh (2005), who found that the effect of testing increases with spaced learning.

Second, from a theoretical perspective, the conceptual terms used by Karpicke and Blunt (2011) seem inaccurate. In the title and throughout the whole paper, they state that retrieval practice is a better learning strategy than concept mapping. However, this terminology is inaccurate as their so-called “retrieval practice” condition actually encompasses not only retrieval practice but also an additional memorization phase. Thus, “retrieval practice” is actually operationalized by a combination of two learning strategies, namely, retrieval practice and memorizing. Therefore, the correct conclusion from Karpicke and Blunt’s (2011) study should be that retrieval practice in combination with additional memorization produces more learning than concept mapping without additional memorization, which accurately reflects their actual operationalization.

Third, from an applied perspective, it seems doubtful that Karpicke and Blunt’s (2011) results can be transferred beyond the laboratory and applied to real-life learning contexts. When preparing for a test where the ability to retrieve memorized facts is measured, it seems unlikely that learning is done as the participants did in Karpicke and Blunt’s (2011) concept mapping condition. The purpose of concept mapping is to structure and organize the content of material that should be learned in order to facilitate its understanding (Novak, 1995; Novak and Cañas, 2006) but not to commit this material to memory for a later memory test. In order to achieve the latter goal, additional memorization strategies beyond establishing a conceptual structure of the text must be used. This is the reason why, according to established text learning techniques such as PQ4R (Thomas and Robinson, 1972), additional activities must follow in order to commit the content to memory so that the content can be successfully retrieved later.

Summing up, the fact that in Karpicke and Blunt’s (2011) study there was an additional memorization phase in the retrieval practice condition but not in the concept mapping condition is problematic from methodological, theoretical, and applied perspectives.

The second potential confounder pertains to the instructions given in Karpicke and Blunt’s (2011) experiment. Here, there is also a critical difference between conditions. In the retrieval practice task, the following instruction was provided above the box where the recalled information had to be typed in: “Please use the space in the box below to write as much information as you can recall about the Sea Otters text you just read” (personal communication with J.R. Blunt). Thus, while performing the retrieval practice task, participants were explicitly prompted that the task was to retrieve and memorize literally everything from the text.

By contrast, in the concept mapping task, the following instruction was provided above the box where the concept map had to be created: “Please use the space below to create your concept map about the Sea Otters text” (personal communication with J.R. Blunt). Only in the instruction provided before it was mentioned “that if they finished [the concept map] before the end of the 25-min period, they should spend the remaining amount of time reviewing their map and making sure they had included all the details from the text in their map” (Karpicke and Blunt, 2011). That is, while performing the concept mapping task, other than in the retrieval practice condition, the participants were not prompted that all information from the text should be included in the created concept map.

Using different instructions, which in one condition but not in the other emphasize that the text should be stored in a way that as much information as possible can be retrieved, may have contributed to the observed difference in the final test performance between the retrieval practice condition and the concept mapping condition. Previous research has shown that the instruction to focus on specific aspects of the learning material while studying can influence the quality of later memory (e.g., McCrudden et al., 2005; Roelle et al., 2015; García-Rodicio, 2023). Therefore, using different instructions in the retrieval practice condition and the concept mapping condition in Karpicke and Blunt (2011) may have led to a different amount of information being processed in the retrieval practice condition vs. the concept mapping condition. Indeed, in Karpicke and Blunt’s (2011) study, descriptively, the proportion of idea units recalled in the retrieval task was higher than the proportion of idea units included in the concept maps (0.81 vs. 0.78). However, given their sample size (20 participants per condition), only large effects can be reliably detected, i.e., *d* > 0.91 with 80% probability. Therefore, it is not possible to assess whether this difference reflects a true effect or not.

Furthermore, concept mapping was not designed as a tool to study as many details of a text as possible but rather as a tool to structure and organize knowledge (Novak, 1995; Novak and Cañas, 2006). Considering that the participants in Karpicke and Blunt’s (2011) concept mapping condition were “instructed about the nature of concept mapping [and] viewed an example of a concept map” (p. 773), it seems likely that the participants viewed concept mapping as a tool to build a mental structure of the relevant contents of a text rather than a tool to foster the ability to later retrieve as much information from the text as possible. Since participants were not prompted in the direction of a potential recall of information during the creation of the concept map, participants’ focus during the creation of the concept map may have been to build the best possible content structure of the text rather than learning all of the details contained in the text. By contrast, the participants in the retrieval practice condition were—while working on the retrieval practice task—explicitly instructed that they should learn the information and details from the text. Since test performance in the final test was mainly determined by the ability to remember as many details from the text as possible, the difference in focus during learning may thus have contributed to the observed advantage of the retrieval practice over the context mapping condition.

In summary, there are two potential confounders in the paradigm used by Karpicke and Blunt (2011) which favor the retrieval practice condition over the concept mapping condition and may thus offer an alternative explanation for the observed performance advantage of the retrieval practice condition over the concept mapping condition. The aim of the present study was to re-examine this issue and to rule out that the reported advantage of retrieval practice over concept mapping in previous studies may actually stem from unnoticed confounders.

To this end, we conducted an experiment which was specifically designed to address the potential confounders as explained above. To avoid the problem of unclear terminology found in previous studies, it is necessary to precisely define the terms used to designate specific cognitive processes. In the present study, “memorizing” is understood as the activity of taking in and storing learning material with the aim of retaining it over a longer period of time in order to be able to recall and reproduce it later. “Retrieval practice” means that participants retrieve previously studied material from memory. “Concept mapping” is understood as the activity of structuring and organizing the content of the learning material in form of a concept map.

**FIGURE 2:**
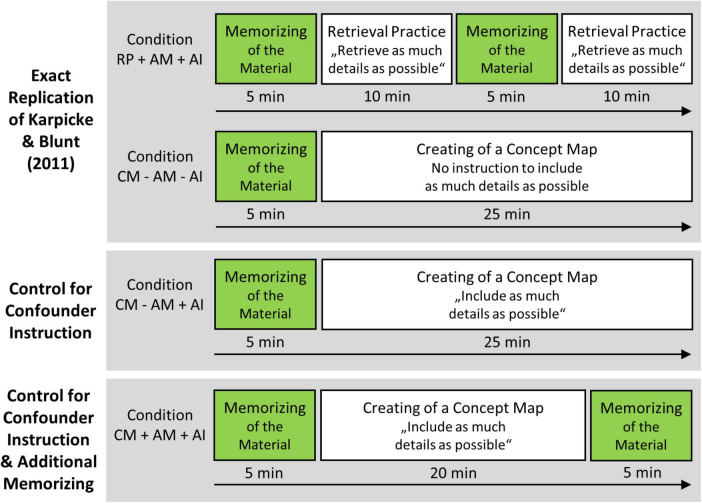
Illustration of the four learning strategy conditions. The “Retrieval Practice with Additional Memorization and with Additional Instruction ‘Recall as Much as Possible’ Condition” (RP + AM + AI) and the “Concept Mapping without Additional Memorization and without Additional Instruction ‘Incorporate as Much as Possible’ Condition” (CM – AM – AI) are exact replications of the conditions examined by Karpicke and Blunt (2011), i.e., RP + AM + AI = Karpicke and Blunt’s “retrieval practice condition”; CM – AM – AI = Karpicke and Blunt’s “concept mapping condition.” In the “Concept Mapping without Additional Memorization and with Additional Instruction ‘Incorporate as Much as Possible’ Condition” (CM – AM + AI), to control for the confounder of different instructions, participants were prompted during the creation of the concept map as well that the concept map should contain as many details of the text as possible. In the “Concept Mapping with Additional Memorization and with Additional Instruction ‘Incorporate as Much as Possible’ Condition” (CM + AM + AI), to additionally control for the confounder of additional memorization, participants were asked to memorize the material after the creation of the concept map as well. Note that the text that the participants were to learn was available during the creation of the concept map but not during retrieval practice.

Besides the exact replication of Karpicke and Blunt’s (2011) original retrieval practice and concept mapping conditions, two additional concept mapping conditions were added (see Figure 2 below). In one condition, to control for the additional memorization in the retrieval practice condition, participants in the concept mapping condition were tasked to memorize the concept map they created, i.e., memorization time in this condition was as long as in the retrieval practice condition, namely 10 min. In the other condition, to control for differences in instructions, participants were instructed during the concept mapping task to create a concept map that contains as many details of the text as possible.

We expected to replicate the findings reported by Karpicke and Blunt (2011), that is, we expected that performance in the final test would be higher in the original retrieval practice condition (with additional memorization) than in the original concept mapping condition (without additional memorization). If the advantage of the retrieval practice condition over the concept mapping condition is actually driven by the additional memorization in the original retrieval practice condition, the advantage of the retrieval practice condition should decrease or even disappear if a second memorization period—after the creation of the concept map—is present. If the advantage of the retrieval practice condition over the concept mapping condition is actually driven by the differences in the instructions used in the original conditions, the advantage of the retrieval practice condition should decrease or even disappear if participants are prompted during the creation of the concept map as well that the concept map should contain as many details of the text as possible.

## 2 Materials and methods

All materials, procedures and statistical tests followed our preregistration at Open Science Foundation^1^ (see).^2^ According to German law, no ethics approval was required as there were no potential negative consequences for the participants of this study.

### 2.1 Participants

A power analysis (G*Power 3.1.9.7; Faul et al., 2007) was used to determine the sample size. Based on a meta-analysis of retrieval practice in the context of teaching by Schwieren et al. (2017), which revealed an overall effect size of *d* = 0.56, the sample size was chosen to be large enough to detect effects of *f* = 0.28 with 95% probability for a one-way ANOVA with four groups (α = 0.05). A total of 240 participants were tested^3^ ; 10 had to be excluded because they were already familiar with the text they were assigned to learn, resulting in a final sample size of *N* = 230. Note that the chosen effect size is more conservative than the effect sizes of *d* = 1.50 found in Karpicke and Blunt (2011) or *d* = 0.96 (verbatim questions) and *d* = 0.62 (inference questions) found by Lechuga et al. (2015), and that the number of participants per condition was about three times higher than in the original study by Karpicke and Blunt (2011).

A total of 227 participants were aged between 18 and 56 (*M*_*Age*_ = 21.65, *SD* = 4.77), 3 stated no age. A total of 178 participants (77.4%) were of female gender, 48 (20.9%) of male gender, and 6 (1.7%) indicated others or no gender. All participants were recruited at the University of Regensburg through bulletins or social media postings. They received either course credit or financial compensation for their participation. All participants provided informed written consent before participating.

### 2.2 Materials

Since the present study was a re-examination of Karpicke and Blunt (2011), the very same materials—translated into German—were employed in this study: The learning material consisted of a text of 277 words (275 in the original English text) on the subject of the sea otter. The final test comprised 16 questions: There were 14 verbatim questions, 12 of which yielded 1 scoring point each, 1 question yielded 2 points and 1 question yielded 7 points, totaling 21 scoring points. Furthermore, there were 2 inference questions, each yielding 2 scoring points. Therefore, a maximum of 25 points in total could be achieved. The answers for these questions were scored identical to Karpicke and Blunt (2011), meaning that only answers which were considered correct in their experiment were considered correct in our study. All other answers were considered false. All answers were rated by two independent raters, whose mutual agreement was very high: They agreed on 4792 out of 4830 scoring points (99.2%) for the verbatim questions. For the inference questions, the raters agreed in 874 out of 920 (95.0%) scoring points. The remaining 38 and 46 cases were solved by discussion until agreement was reached. The result of the final test is given as percentage of the maximum possible score, i.e., 21 points for the verbatim questions and 4 points for the inference questions.

### 2.3 Procedure

A one-by-four between-subjects design was employed, with learning strategy in combination with potential confounders as factor and the following conditions as factor levels: retrieval practice with additional memorization and with additional instruction “recall as much as possible” (RP + AM + AI condition; original retrieval practice condition as in Karpicke and Blunt, 2011), concept mapping without additional memorization and without additional instruction “incorporate as much as possible” (CM - AM - AI condition; original concept mapping condition as in Karpicke and Blunt, 2011), concept mapping without additional memorization and with additional instruction “incorporate as much as possible” (CM - AM + AI condition), and concept mapping with additional memorization and with additional instruction “incorporate as much as possible” (CA + AM + AI condition).

The experiment consisted of two sessions conducted in person. In the learning session, participants studied the learning material according to different learning strategies. One week later, in the testing session, participants answered the final test (identical to Karpicke and Blunt, 2011). Participants were tested in groups of up to four persons, although each participant had their own individual, separate cubicle.

At the beginning of the experiment, all participants received general written instructions that they were to learn a text and that they would be tested 1 week later. The instructions stated that all information from the text should be memorized. In all conditions, participants were given the appropriate timeframe of the particular condition (see below). In the three concept mapping conditions, participants were also given a short written instruction, including a graphic example, on the nature of concept maps and how concept maps work. Although Mintzes et al. (2011) criticized Karpicke and Blunt (2011) on the grounds that working with concept maps must be learned thoroughly over a longer period of time and cannot be taught *ad hoc* by means of a short instruction, our focus here lies on the methodology of Karpicke and Blunt’s (2011) experiment. Thus, even if studying with concept mapping is more efficient with more experience (see also Lechuga et al., 2015), the methodology of the experiment would not be affected. Hence, we retained Karpicke and Blunt’s (2011) original procedure.

For the learning session, the overall duration of the learning phase was 30 min in all conditions. In all conditions, participants initially had 5 min to study the text (identical to Karpicke and Blunt, 2011). After this point, the conditions differed: In the RP + AM + AI condition, the text was removed in the first recall phase and participants were asked to write down as much as they could recall from the text they just learned. They were given 10 min for this task before they memorized the text once more for a period of 5 min, followed by a second recall phase of 10 min. In the CM – AM – AI condition, participants kept the text for the whole duration of the studying time; participants in this condition then had 25 min to create their concept map on a sheet which simply stated that the concept map should be created below. In the CM – AM + AI condition, the text was also left with the participants for the whole time, who also had 25 min to create their concept map. However, in this condition, the instruction on the sheet for the concept map explicitly stated that the concept map should be created below and that as much information as possible from the text should be incorporated in doing so. This instruction was analogous to the instruction in the retrieval practice condition for the retrieval practice task, which stated that the participants should recall as much information as possible. In the CM + AM + AI condition, the text was also left with the participants, who then had 20 min to create their concept maps. The instruction on the sheet for the concept map stated that the concept map should be created below and that as much information as possible from the text should be incorporated in doing so. After 20 min, the participants were asked to memorize the concept maps they had just created for 5 min.

Afterward, in all four conditions, all participants filled out a questionnaire on metacognitive and demographic questions, which employed the very same items and scales as Karpicke and Blunt (2011).

The testing session, 1 week after the learning session, was identical for all four conditions: All participants were given the final test, i.e., the 14 verbatim and 2 inference questions. The time for the final test was not limited, which is identical to the procedure of Karpicke and Blunt (2011).

## 3 Results

**FIGURE 3:**
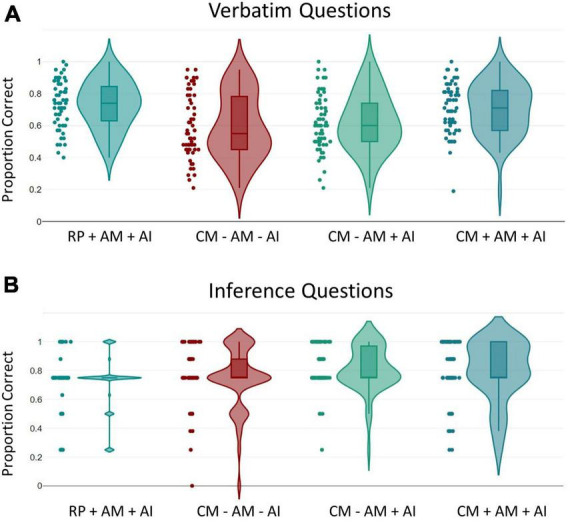
Memory Performance. The proportion of correct answers for verbatim questions **(A)** and inference questions **(B)** is shown as a function of the four learning strategy conditions (Retrieval Practice with Additional Memorization and with Additional Instruction “Recall as Much as Possible,” RP + AM + AI; Concept Mapping without Additional Memorization and without Additional Instruction “Incorporate as Much as Possible,” CM – AM – AI; Concept Mapping without Additional Memorization and with Additional Instruction “Incorporate as Much as Possible,” CM – AM + AI; Concept Mapping with Additional Memorization and with Additional Instruction “Incorporate as Much as Possible,” CM + AM + AI). The violin plots show the probability density across participants; data points are plotted as dots. Center horizontal line markers show the medians. Box limits indicate the 25th and 75th percentiles. Whiskers extend 1.5 times the interquartile range from the 25th and 75th percentiles.

The proportion of correct answers for the verbatim questions and the inference questions in the final test as a function of experimental condition is shown in Figure 3 below.

For the verbatim questions, an analysis of variance (ANOVA) with the factor of learning strategy condition (RP + AM + AI condition vs. CM – AM – AI condition vs. CM – AM + AI condition vs. CM + AM + AI condition) revealed a significant effect, *F*_(3, 226)_ = 8.33, *p* < 0.001, η_*p*_^2^ = 0.10.

*Post-hoc* comparisons using the Tukey’s HSD test indicated that performance was significantly higher (*p* < 0.001) in the RP + AM + AI condition (*M*_*RP + AM + AI*_ = 0.73, *SD* = 0.15) than in the CM – AM – AI condition (*M*_*CM* – *AM* – AI_ = 0.59, *SD* = 0.20). However, performance did not differ (*p* = 0.854) between the RP + AM + AI condition and the CM + AM + AI condition (*M*_*CM + AM + AI*_ = 0.70, *SD* = 0.16), indicating that the advantage of the retrieval practice condition disappeared when the same instruction was used in the concept mapping condition and when memorization time was equal. The CM – AM + AI condition (*M*_*CM* – *AM + AI*_ = 0.62, *SD* = 0.18) was outperformed by both the RP + AM + AI condition (*p* = 0.004) and the CM + AM + AI condition (*p* = 0.041). The CM – AM – AI condition was outperformed by the CM + AM + AI condition (*p* = 0.003) but not by the CM – AM + AI condition (*p* = 0.842), indicating that the instruction does not play a decisive role.^4^

For the inference questions, an ANOVA revealed a significant effect as well, *F*_(3, 226)_ = 2.99, *p* = 0.032, η_*p*_^2^ = 0.038. Descriptively, performance was higher in all concept mapping conditions (*M*_*CM* – *AM* – AI_ = 0.75, *SD* = 0.21; *M*_*CM* – *AM + AI*_ = 0.81, *SD* = 0.15; *M*_*CM + AM + AI*_ = 0.79, *SD* = 0.21) compared to the retrieval practice condition (*M*_*RP + AM + AI*_ = 0.71, *SD* = 0.19). *Post-hoc* comparisons using the Tukey’s HSD test indicated that the only statistically significant difference (*p* = 0.038) was between the RP + AM + AI and the CM – AM + AI condition. All other differences between conditions were not significant (all *p*s > 0.111). A comparison of the retrieval practice condition vs. all concept mapping conditions (collapsed data: *M*_*CM*_ = 0.78, *SD* = 0.19) showed that the performance in the retrieval practice condition was significantly lower, *t*(228) = 2.41, *p* = 0.008, *d* = 0.36.

To rule out that previous experience with the learning strategy, i.e., with retrieval practice or concept mapping, might have influenced the results, participants’ previous experience was examined. The percentage of participants indicating that they had previous experience with the learning strategy they employed was higher in the retrieval practice condition (58.2%) compared to the concept mapping conditions (CM – AM – AI condition: 27.6%; CM – AM + AI condition: 34.5%; CM + AM + AI condition: 33.9%), *F*_(3,226)_ = 4.46, *p* = 0.005, η_*p*_^2^ = 0.056. Previous experience with the learning strategy in the retrieval practice condition was higher compared to each of the individual concept mapping conditions (all *p*s < 0.043), which did not significantly differ from each other (all *p*s > 0.864), according to a Tukey’s HSD *post-hoc* test. For both the verbatim and the interference questions, a four-by-two ANOVA with the between subjects factors of learning strategy (RP + AM + AI condition vs. CM – AM – AI condition vs. CM – AM + AI condition vs. CM + AM + AI condition) and previous experience with the learning strategy (previous experience vs. no previous experience) indicated neither a significant main effect of previous experience with the learning strategy [verbatim questions: *F*_(1, 222)_ = 0.12, *p* = 0.733, η_*p*_^2^ = 0.001; inference questions: *F*_(1, 222)_ = 0.21, *p* = 0.651, η_*p*_^2^ = 0.001] nor a significant interaction [verbatim questions: *F*_(3, 222)_ = 0.24, *p* = 0.871, η_*p*_^2^ = 0.003; inference questions: *F*_(3, 222)_ = 1.74, *p* = 0.160, η_*p*_^2^ = 0.023].

Furthermore, we examined previous knowledge about sea otters, assessment of text difficulty, and interest in the text to rule out that these factors may have influenced the results. There were neither statistically significant differences between the learning strategy conditions for previous knowledge on sea otters, *F*_(3, 226)_ = 2.44, *p* = 0.065, η_*p*_^2^ = 0.023, nor for text difficulty, *F*_(3, 226)_ = 0.49, *p* = 0.690, η_*p*_^2^ = 0.006, nor for interest in the text, *F*_(3,226)_ = 0.03, *p* = 0.992, η_*p*_^2^ < 0.001.

Concerning the judgments of learning, an ANOVA revealed a significant effect as well, *F*_(3, 226)_ = 10.22, *p* < 0.001, η_*p*_^2^ = 0.12. *Post hoc* comparisons using the Tukey’s HSD test indicated that judgment of learning in the RP + AM + AI condition (*M* = 44.18, *SD* = 16.30) was significantly lower than in the CM – AM – AI condition (*M* = 54.10, *SD* = 16.86; *p* = 0.005), the CM – AM + AI condition (*M* = 59.14, *SD* = 17.09; *p* < 0.001), and the CM + AM + AI condition (*M* = 58.64, *SD* = 14.68; *p* < 0.001). This replicates previous findings, showing that participants’ assessment of how much they would remember 1 week later is significantly lower in the retrieval practice condition (e.g., Roediger and Karpicke, 2006; Karpicke and Blunt, 2011; but see Weissgerber and Rummer, 2023, for a critical discussion of judgements of learning in the context of retrieval practice).

## 4 Discussion

Our results clearly show that the memory advantage in the retrieval practice condition over the concept mapping condition reported in Karpicke and Blunt (2011) and, by extension, also in Lechuga et al. (2015), Camerer et al. (2018), and O’Day and Karpicke (2021), who employed the very same methodology, does in fact not prove that retrieval practice produces more learning than studying with concept mapping. When controlling for the methodological problem in these studies—namely that there was an additional memorization phase in the retrieval practice condition—the advantage of retrieval practice over concept mapping disappeared.

Concerning the verbatim questions, our data replicated Karpicke and Blunt’s (2011) finding that performance in a retrieval practice condition where participants additionally memorize the learning material is better compared to a concept mapping condition without additional memorization. However, when participants also additionally memorize the learning material in the concept mapping condition, there is no statistically significant difference in performance between retrieval practice and concept mapping.

This finding indicates that Karpicke and Blunt’s (2011) results were actually driven by the additional memorization in the retrieval practice condition rather than by differences inherent to the respective learning strategies, i.e., retrieval practice and concept mapping. The relevant role of memorization is further corroborated by the finding that performance in both conditions with additional memorization (RP + AM + AI and CM + AM + AI) was also better compared to the condition without additional memorization but where participants were instructed during the concept mapping task to cover as much information from the text as possible (CM – AM + AI). This represented another potential confounding factor in the study by Karpicke and Blunt (2011). The finding that performance in the concept mapping conditions without additional memorization did not differ as a function of the instruction provided during the concept mapping task indicates that the difference in the instruction does not play an important role for performance and is—at least in this setting—probably not a confounder.

Concerning the inference questions, the situation is entirely different from the verbatim questions. In contrast to Karpicke and Blunt (2011)—and to Lechuga et al. (2015) and O’Day and Karpicke (2021) as well—we unexpectedly found that the performance in the retrieval practice condition was lower than in the concept mapping conditions. As there were no significant differences in performance between the concept mapping conditions, neither the difference in the instruction nor—more importantly—in memorization seems to affect performance on the inference questions. However, from the perspective of classical test theory, measuring a highly complex construct such as meaningful learning with a diagnostic instrument consisting of merely two questions (or four scoring points) seems hardly adequate as very short test lengths negatively affect both reliability and validity (e.g., Novick, 1966; McDonald, 2013; Hogan, 2019). Thus, any conclusion drawn from such basis can only be tentative and must be taken with a pinch of salt.

In the present study, previous experience with concept mapping was lower than in Karpicke and Blunt’s (2011) study. Lechuga et al. (2015) found that memory performance increased when participants were already familiar with and frequently used concept mapping compared to participants who had no experience in concept mapping and were trained for the purpose of the experiment. Accordingly, if the participants of the concept mapping condition in the present experiment had had a similar level of prior experience with concept mapping as in Karpicke and Blunt’s (2011) study, their performance might have been even higher. In an applied context, this suggests that training in concept mapping and experience through regular application could improve performance, as already suggested by Mintzes et al. (2011).

The present study is mainly concerned with the methodology behind experiments comparing retrieval practice and concept mapping as learning strategies. However, the finding that the previously reported advantage of retrieval practice is actually driven by a confounder, i.e., by a different amount of memorization rather than by differences between the learning strategies of retrieval practice and concept mapping, has far-reaching consequences beyond methodology, which can only be touched upon here.

Concerning cognitive psychology, the advantage observed in previous studies of the retrieval practice condition over the concept mapping condition was explained by, for instance, the decisive role of better cue diagnosticity (Karpicke and Blunt, 2011) or active “access [to] already encoded information in memory” (Lechuga et al., 2015, p. 61). However, the present study now shows how the advantage of the retrieval practice condition observed in previous studies actually stemmed from additional memorization which was present in the retrieval practice condition but not in the concept mapping condition. Since the advantage of retrieval practice over concept mapping disappears when participants in the concept mapping condition, too, memorize, it seems to be the case that cognitive processes related to retrieval practice (such as cue diagnosticity or active access to already encoded information in memory) do not to improve memory, at least when studying textbook contents with elaborative learning strategies. In fact, this is in line with the results of a recent meta-analysis of the testing effect in classroom learning by Yang et al. (2021) who found virtually no advantage (Hedges’ *g* = 0.095) of retrieval practice over various forms of elaborative learning strategies.

Concerning educational practice, the finding that the advantage of retrieval practice over concept mapping observed in previous studies is actually a methodological artifact challenges current recommendations for learning in real-life contexts. Based on their methodologically flawed findings, Karpicke and Blunt (2011), for instance, conclude that the human mind supposedly works in a way “that differs from everyday intuition” (p. 774) and that their finding may “pave the way for the design of new educational activities based on consideration of retrieval processes” (p. 774). In the light of the present findings, however, such conclusions seem invalid. When appropriately controlling for confounding factors in the previous studies, retrieval practice and concept mapping seem equally effective in promoting memory performance. However, it should be noted that the effectiveness of different learning strategies may vary as a function of the length of the retention interval, as suggested, for example, by the finding that the testing effect depends on the retention interval (e.g., Halamish and Bjork, 2011; Kornell et al., 2011; for a review, see Rowland, 2014). In Karpicke and Blunt’s (2011) study as well as the present study retention intervals of 1 week were used so that equal effectiveness of retrieval practice and concept mapping, as observed in the present study, was demonstrated only for a retention interval of 1 week. Therefore, further research is needed to investigate whether the present findings also apply to other retention intervals.

The aim of the present study was to examine whether the memory advantage in the retrieval practice condition over the concept mapping condition, as observed in the paradigm developed by Karpicke and Blunt (2011), is actually not driven by retrieval practice itself but rather by the confounding variables of an additional memorization phase and the constantly visible instruction to retrieve as many details from the text as possible in the retrieval practice condition. The results clearly showed that the memory advantage observed in Karpicke and Blunt’s (2011) paradigm indeed stems from these confounding variables because the advantage disappeared when the concept mapping condition also included—as was the case in the retrieval practice condition—an additional memorization phase and a constantly visible instruction to include as much information as possible from the text in the concept map. While the results of the present study clearly answered the research question for which it was designed, the results raise further questions for future research.

For instance, it is important to note that the additional memorization in the retrieval practice condition differed from the additional memorization in the concept mapping condition in one respect. In the retrieval practice condition, participants were asked to memorize the text again after retrieval practice, while in the concept mapping condition they were asked to memorize the concept map they had created. From an applied perspective, this makes sense because first studying the text by creating a concept map, but then putting that created concept map aside and then going back to the text to study for the upcoming test invalidates the idea of using the concept map to learn the text. Similarly, it would hardly make sense to provide participants in the retrieval practice condition with a concept map after retrieval practice and to ask them now to memorize the concept map instead of the text for the upcoming test. Therefore, from an applied perspective, it is important that the type of material memorized matches the appropriate learning strategy to ensure ecological validity.

However, from the perspective of basic experimental psychology, where the goal is to investigate basic cognitive mechanisms independent of applied contexts, it is interesting to see whether it makes a difference if participants additionally memorize either the text or the created concept map after having created a concept map. Interestingly, in a study by O’Day and Karpicke (2021), participants, after having created a concept map, performed a memorization task where they were asked to use the text for memorization and a retrieval task where they were asked to retrieve the contents of the text. The results of O’Day and Karpicke’s (2021) Experiment 2, where the same concept mapping task was used as in our study, were fully consistent with the present results: Retrieval practice combined with additional memorization (so-called “retrieval practice” condition) only outperformed concept mapping when participants performed a concept mapping task without additional memorization and retrieval but not when participants additionally memorized and retrieved the text after the creation of the concept map. This learning activity, after having created the concept map, was a combination of text memorization and retrieval practice. Therefore, it is an interesting question for further basic research whether additional memorization of the text alone after a concept mapping task improves memory as well.

Similarly, it is important to note that the retrieval practice task and the concept mapping task differed in one aspect in the present study: in the retrieval practice task, the text the participants were to learn was not available, whereas, in the concept mapping task, the text was available. Again, from an applied perspective, this is reasonable because retrieval practice hardly makes sense when the text is available, or conversely, creating a concept map hardly makes sense when the text is not available. However, again from a basic experimental psychology perspective where research questions are not necessarily investigated with a focus on their applicability in real life, it would be interesting to examine what happens when retrieval practice is performed with the text being avaible, or conversely, when a concept map is created without the text being available. Indeed, the question of what happens when participants create a concept map without the text being available was already addressed in a previous study by Blunt and Karpicke (2014) and their results are fully consistent with the results of the present study. There, retrieval practice without the text being available in combination with additional memorization (so-called “retrieval practice” condition) did not outperform concept mapping without the text being available in combination with additional memorization (so-called “retrieval-based concept mapping” condition; Blunt and Karpicke, 2014).

These differences between the perspectives of applied and basic research, as presented in the preceding paragraphs, draw attention to the sometimes overlooked fact that the research logics of basic and applied research differ. Although the domains of real-life learning and experimental research overlap, their underlying rationalities diverge (e.g., Goldthorpe, 2001). From the perspective of basic experimental research, comparing specific learning conditions in isolation or comparing all possible combinations of learning conditions makes perfect sense, regardless of their relevance to applicability. However, such a research strategy does not necessarily make sense from the perspective of applied research as well because not all learning conditions that can be isolated or (re-)combined in different ways in the laboratory are feasible in real-life learning.

**FIGURE 4:**
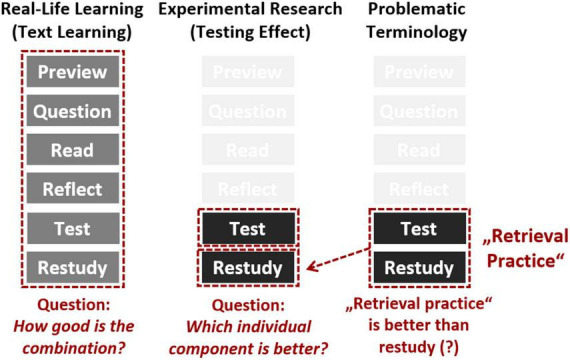
Illustration of the divergent rationalities underlying real-life learning and experimental research. Although the domains overlap, the focus of the questions asked is different: determining the optimal combination of cognitive processes (real-life learning) vs. determining the specific effect of isolated cognitive processes (experimental research). As shown on the right side, this problem may be obfuscated by the use of imprecise terminology. If the term “retrieval practice” is used to delineate a learning strategy which is actually a combination of retrieval practice and restudying, this may lead to results that may seem surprising and informative (e.g., “retrieval practice is better than restudying”) at first glance, although they are actually rather trivial (e.g., “retrieval practice plus restudying is better than restudying alone”). Consequently, potential implications for education drawn on the basis of experimental laboratory studies should be considered with caution as overemphasizing one factor or an oversimplified transfer to real-life learning may lead to already existing knowledge on learning being neglected.

This case is illustrated in Figure 4 below. From a basic experimental perspective, the finding that (isolated) testing is more effective than (isolated) restudying is interesting and informative because it shows that different mental activities affect later memory performance differently. However, from an applied perspective, such a finding is less informative because in real-life learning, optimal studying actually comprises the combination of different learning strategies, including both testing and restudying, as reflected both in well-known study methods such as PQ4R (Thomas and Robinson, 1972) and in students’ real-life learning behavior (Hartwig and Dunlosky, 2012; Blasiman et al., 2017; Kuhbandner and Emmerdinger, 2019). In particular, as illustrated in Figure 4 (on the right side), this problem may be obfuscated by the use of imprecise terminology. If the term “retrieval practice” is used to delineate a learning strategy which is actually a combination of retrieval practice and restudying, this may lead to results that may seem surprising and informative (e.g., “retrieval practice is better than restudying”) at first glance, although they are actually rather trivial (e.g., “retrieval practice plus restudying is better than restudying alone”). Thus, potential implications for education drawn on the basis of experimental laboratory studies should be considered with caution as overemphasizing one factor or an oversimplified transfer to real-life learning may lead to already existing knowledge on learning being neglected.

On a more general level, this study further demonstrates that it is essential in research to describe theoretical concepts and the related operationalizations in appropriate terminology. When investigating a complex topic such as learning strategies, which involve a variety of mental processes in different contexts, it is necessary to clearly define and delineate different learning strategies from one another so that unambiguous and valid conclusions can be drawn. As shown in the present study, if the terms used to communicate a finding do not exactly reflect what participants actually did, invalid conclusions can be drawn. Although Karpicke and Blunt’s (2011) retrieval practice condition included an additional second learning strategy, i.e., memorization, the authors did not account for this at the conceptual-linguistic level because they make general statements about retrieval practice and concept mapping as learning strategies. In other words, their terminology blurs and confuses what was actually done in their experiment. Thus, their conclusion that retrieval practice produces more learning than concept mapping—prominently featured in the title of their study—is both invalid and inaccurate in this generalized form and therefore misleading. In fact, similar problems at the level of terminology are found in other studies on retrieval practice as well, as shown, for instance in a recent study on the use of misleading terms in questionnaire studies on the use of retrieval practice in real-life learning (Kuhbandner and Emmerdinger, 2019).

In conclusion, by demonstrating that the advantage of retrieval practice over concept mapping observed in previous studies was actually driven by an additional memorization period in the retrieval practice condition, the present study serves as a reminder of the importance of a solid methodology. Furthermore, the present study also illustrates the importance of employing precise terms and language which precisely reflect—in both directions—the relation of theoretical concepts and actual operationalization. On a more general level, the present findings illustrate that one should be cautious when transferring experimental findings to real life learning contexts and be aware of the divergent rationalities underlying experimental research and educational practice.

## Data availability statement

The original contributions presented in this study are publicly available. This data can be found here: https://osf.io/fj95g/.

## Ethics statement

Ethical approval was not required for the studies involving humans because According to German law, no Ethics approval was required as there were no potential negative consequences for the participants of this study. The studies were conducted in accordance with the local legislation and institutional requirements. The participants provided their written informed consent to participate in this study.

## Author contributions

RM: Conceptualization, Writing – original draft, Writing – review and editing. CK: Conceptualization, Writing – review and editing. KF: Writing – review and editing.

## Footnotes

1 As outlined in the preregistration, this experiment further encompassed additional, new inference questions. The results will be addressed in a future article as this article focuses on methodology, based on an exact replication of Karpicke and Blunt (2011). The participants answered those additional inference questions on a separate sheet after completing the original experiment and test from Karpicke and Blunt’s (2011) study. Hence, the findings reported here were not influenced by the additional inference questions.

2
https://osf.io/zx7h2

3 The preregistration stated that the target sample was *N* = 160. This number was increased since statements about the absence of an effect are more reliable when the power is higher, and we chose a sample size that allows the detection of a possible effect with 95% probability, i.e., *N* = 224.

4 There was one extreme outlier in the CM + AM + AI condition (more than 3 SD below the mean; see also Figure 3). Removing this outlier, the results are as follows: *F*_(3, 225)_ = 9.17, *p* < 0.001, η_p_^2^ = 0.11. The appropriate *post hoc* comparisons are: CM + AM + AI (*M*_CM +_ _AM +_ _AI_ = 0.71, *SD* = 0.15) vs. RP + AM + AI: *p* = 0.950; vs. CM – AM + AI: *p* = 0.017; vs. CM – AM – AI: *p* < 0.001.
